# Natural Product and Natural Product-Derived Gamma Secretase Modulators from *Actaea Racemosa* Extracts

**DOI:** 10.3390/medicines2030127

**Published:** 2015-06-30

**Authors:** Mark A. Findeis, Frank C. Schroeder, Steffen P. Creaser, Timothy D. McKee, Weiming Xia

**Affiliations:** 1 Aria Neurosciences, Incorporated, 295 Washington Ave, Suite 4N, Hamden, CT 06518, USA; 2 Boyce Thompson Institute and Department of Chemistry and Chemical Biology, Cornell University, 533 Tower Road, Ithaca, NY 14853-1801, USA; E-Mail: schroeder@cornell.edu; 3 Genzyme, 500 Kendall Street, Cambridge, MA 02142, USA; E-Mail: steffen.creaser@gmail.com; 4 Biogen, 115 Broadway Street, Cambridge, MA 02142, USA; E-Mail: timothy.mckee@biogen.com; 5 Department of Veterans Affairs, ENR Memorial Veterans Hospital, 200 Springs Road, Bedford, MA 01730, USA; E-Mail: weiming.xia@va.gov

**Keywords:** Alzheimer’s disease, amyloid precursor protein, beta-amyloid, gamma secretase modulator, *Actaea racemosa*, black cohosh, natural product, SPI-014, SPI-1865

## Abstract

Alzheimer’s disease is characterized by pathogenic oligomerization, aggregation, and deposition of amyloid beta peptide (Aβ), resulting in severe neuronal toxicity and associated cognitive dysfunction. In particular, increases in the absolute or relative level of the major long form of Aβ, Aβ42, are associated with increased cellular toxicity and rapidity of disease progression. As a result of this observation, screening to identify potential drugs to reduce the level of Aβ42 have been undertaken by way of modulating the proteolytic activity of the gamma secretase complex without compromising its action on other essential substrates such as Notch. In this review we summarize results from a program that sought to develop such gamma secretase modulators based on novel natural products identified in the extract of *Actaea racemosa,* the well-known botanical black cohosh. Following isolation of compound **1** (SPI-014), an extensive medicinal chemistry effort was undertaken to define the SAR of **1** and related semisynthetic compounds. Major metabolic and physicochemical liabilities in **1** were overcome including replacement of both the sugar and acetate moieties with more stable alternatives that improved drug-like properties and resulted in development candidate **25** (SPI-1865). Unanticipated off-target adrenal toxicity, however, precluded advancement of this series of compounds into clinical development.

## 1. Introduction

Alzheimer’s disease (AD) is the most common type of dementia and affects approximately 5.3 million people in the United States where it is the sixth leading cause of death [1]. Worldwide incidence is approximately 44 million [2] and is projected to increase substantially over the next several decades. Because of the high morbidity of individuals suffering from AD, the cost of care for this increasing cohort of patients is extraordinarily high. Accounting for both direct and indirect costs for care of AD patients’ results in a combined annual cost in the United States alone of over $430 billion [1]. Given this enormous societal burden, there is a great need for effective therapies for AD that can slow or halt the progression of this disease. Currently approved drugs for AD target the symptoms and do not address the underlying mechanisms of disease.

Alois Alzheimer was the first to associate the brain pathology of the disease that was to be named after him with the symptoms of dementia [3,4]. It was another three quarters of a century before the molecular understanding of AD began to be established with the discovery of amyloid precursor protein (APP) as the source of amyloid beta-peptide (beta-amyloid, Aβ) [5]. The ensuing elucidation of the processing of APP by beta and gamma secretases to produce the variably sized Aβ of primarily 40 or 42 residues in length resulted in the “Amyloid Hypothesis” of AD [6,7,8] stating that AD is directly tied to the aggregation, deposition and toxicity of Aβ. Subsequent research has provided further evidence that the long form of Aβ (Aβ42) is particularly associated with the onset and progression of AD [9].

The gamma secretase enzyme complex variably cleaves APP at the position that determines the carboxyl terminus of Aβ. Early screening programs that used cell-based assays that targeted the reduction of the production of Aβ, but before the processing of APP was well defined, generally identified small molecule gamma secretase inhibitors (GSIs) which potently lowered Aβ levels. The subsequent identification of the multi-component gamma secretase complex and the demonstration of its essential role in development raised doubts about whether a GSI could be used safely to treat AD. Serious mechanism-based side effects were indeed subsequently observed in human clinical trials. To address this challenge, the concept of selective gamma secretase inhibitors or gamma secretase modulators (GSMs) was developed with the goal of reducing production of Aβ, and Aβ42 in particular, while allowing otherwise normal processing of other substrates of gamma secretase. This type of selectivity appeared to be possible based on the observation that certain non-steroidal anti-inflammatory drugs had GSM activity [10]. In follow-up to this initial report, a subsequent unbiased screening study was undertaken with the goal of identifying an already approved drug or a nutritional supplement with GSM activity [11]. Among a total sample set of approximately 2000 individual compounds and mixtures of compounds, including previously identified GSMs, a single sample was identified with robust and potent selective Aβ42 lowering activity well below a concentration that resulted in any cellular toxicity [12]. This one sample was a standard alcoholic extract of the root and rhizome of *Actaea racemosa*, the black cohosh plant.

Black cohosh extract (BCE) is a well-known nutritional supplement used in traditional folk medicine as an anti-inflammatory agent and is now used as an herbal remedy for treatment of hot flashes during menopause [13,14]. It is currently cultivated and wild-harvested internationally to support the commercial market, including major supplies from Europe, North America and Asia [15,16]. Despite this broad use, the isolation and physical characterization of the components of this plant was relatively limited and the study of the pharmacology of its main components was further limited. The observation of selective Aβ42 lowering activity resulted in the identification of new compounds not previously observed in black cohosh or other plants, the preparation of many modified derivatives of these compounds, and extensive *in vitro* and *in vivo* characterization of their pharmacological properties.

## 2. Novel Gamma Secretase Modulators Based on Black Cohosh

### 2.1. Isolation of 1 (SPI-014) from Black Cohosh

**Figure 1:**
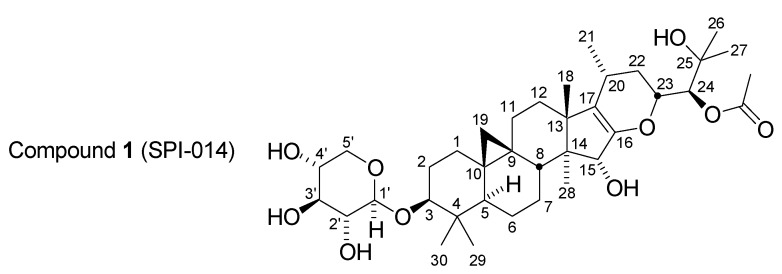
Structure of Compound 1 (SPI-014).

The initial observation of Aβ42 lowering activity in BCE was obtained via the assay of a sample of liquid BCE purchased as a nutritional supplement at a retail store. Such commercial retail products, however, are typically diluted with co-solvents and additives including glycerin and propylene glycol which hinder analysis of the components of the extract. To support a more detailed fractionation of the components of BCE, samples of semi-solid “raw” extract were obtained from commercial production sources. These materials are typically prepared by bulk percolation of powdered dried root-rhizome of the plant with an ethanol-water mixture. Evaporation of solvent affords the crude extract. This material was easier to resuspend in an appropriate manner to allow subsequent fractionation by normal phase chromatography. Using a combination of normal phase chromatography on silica gel and iterative reverse-phase HPLC in combination with a cell-based assay for production of Aβ40 and Aβ42, a set of nine compounds was isolated [17]. Three of these compounds were isolated by crystallization directly from the silica gel fractions: actein, and the xylopyranoside and arabinopyranoside of cimigenol. These compounds, present in relatively large amounts, were previously known constituents of BCE and were inactive. Further bioassay-guided fractionation by HPLC, however, afforded a series of three isomeric shengmanol glycosides and their corresponding and not previously observed enol ether derivatives. One of these compounds, **1** (Figure 1),

24(*S*)-*O*-acetylhydroshengmanol (Δ-16,17)-enol ether-3-*O*-β-D-xylopyranoside (also named SPI-014; CAS Registry Number 915277-86-0: β-D-Xylopyranoside, (3β,15α,23*R*,24*S*)-24-(acetyloxy)-16,23-epoxy-15,25-dihydroxy-9,19-cyclolanost-16-en-3-yl), was found to be a potent and selective inhibitor of Aβ42 production (IC_50_ of 100 nM) in comparison to its effect on Aβ40 (IC_50_ of 63 µM) [17].

Further characterization of **1** found that it could lower Aβ42 levels while allowing otherwise normal processing of APP to occur as measured by the production of other major proteolytic fragments of APP. Compound **1** was also found to be active *in vivo*, achieving selective reduction of Aβ42 relative to Aβ40 in the brains of normal mice. In addition to selective reduction of Aβ42 relative to Aβ40, immunoprecipitation mass spectrometric (IP-MS) analysis of all lengths of Aβ in cell conditioned medium revealed a novel change in the distribution of Aβ(1-x) lengths. Not only did the proportion of Aβ42 go down but the proportions of other shorter length Aβs, Aβ(1-37) and Aβ(1-39), were increased. This result suggested that **1** could produce a shift in the overall average length of the Aβ pool to shorter, less pathogenic forms [9,17]. Additional preliminary screening for unwanted off-target pharmacology also indicated an absence of interaction with a range of targets including steroid and hormone receptors [17], consistent with prior reports that BCE possessed no apparent direct estrogen receptor modulation activity [18,19,20,21,22]. Compound **1** thus became a lead compound of considerable interest for possible elaboration into a drug candidate suitable for testing as a potential treatment for AD [17].

### 2.2. Preliminary Structure–Activity Relationships of 1 (SPI-014)

**Figure 2:**
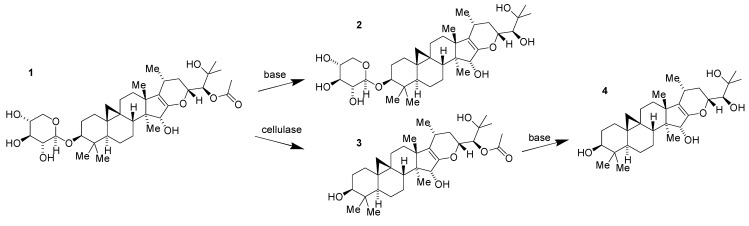
Conversion of **1** to compounds **2**, **3** and **4**.

Limitations of **1** as a potential drug candidate in its own right were suggested immediately by its structure. The presence of the labile *O*-glycoside and acetate moieties was anticipated to create unwanted chemical and biochemical instability. This expectation was promptly confirmed by pharmacokinetic studies in mice showing rapid conversion of **1** to its des-acetate **2**, aglycone **3** and des-acetate aglycone **4** in plasma and brain [17]. All three metabolites were prepared as derivatives of **1** (Figure 2) and also found to have substantially less bioactivity than the parent compound. The medicinal chemistry challenge that was thus presented was how to retain, if not enhance, the potency of the parent compound in a metabolically robust derivative or analog.

**Figure 3:**
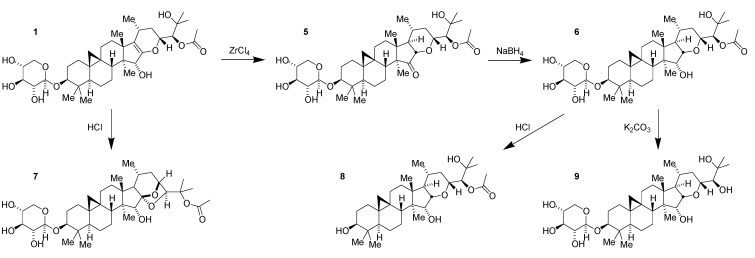
Conversion of compound **1** to compounds **5~9**.

As part of efforts to understand the structure–activity relationships (SAR) of **1** further, an effort was made to reduce the enol ether. Direct reduction proved to be difficult. Following screening of a variety of acid-catalyzed conditions, it was found that catalytic ZrCl_4_ in CH_2_Cl_2_ would facilitate the rearrangement of the enol ether to the 15-keto tetrahydropyran **5** (Figure 3) [23]. Subsequent stereoselective reduction obtained the net reduction of the enol ether with trans-fused stereochemistry. The resulting alpha-hydroxy tetrahydropyran **6** proved to be a useful compound in several regards.

While the ketone **5** was moderately less potent than its parent compound, recapturing the alcohol **6** following reduction more than regained the potency of the parent compound indicating that the trans-fused tetrahydropyran provided a gain in activity and was a promising scaffold for further chemistry. In addition, the increased stability of the tetrahydropyran allowed more facile and selective manipulation for the synthesis of additional compounds. Previously, acid treatment of **1** had resulted in rearrangement to form the 24-*O*-acetyl cimigenol **7** (Figure 3). This intramolecular cyclization is no longer possible in **6**, and, as a result, it is possible to remove the xylose selectively to obtain the aglycone **8** (Figure 3) without the need to resort to the use of milder enzymic catalysis for sugar removal as is required to obtain the aglycone of **1** [17]. As before with the enol ether, mild base removes the acetate to generate the 1,2-diol **9** (Figure 3). And, as before with **1**, these derivatives of the tetrahydropyran have less activity than the parent acetate glycoside **6**.

**Figure 4:**
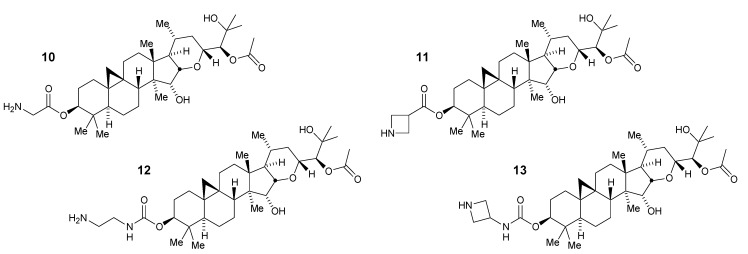
3-*O*-Modified ester and carbamate derivatives of aglycone **8**.

With the aglycone **8** of the acetate **6** in hand it was possible to explore substitutions at the 3-*O*-hydroxyl position through the synthesis of C3 esters and carbamates [23]. In the ester series, installation of the sterically compact glycyl and azetidine-3-carboxyl groups in place of xylose afforded potent and selective compounds **10** and **11**, respectively (Figure 4).

These observations were repeated in the more stable carbamate series in which the 2-aminoethyl and 3-azetidinyl derivatives provided potent compounds **12** and **13** (Figure 4). Interestingly, the selectivity for Aβ42 over Aβ40 was diminished in this latter compound to less than 10-fold (47 nM *vs*. 330 nM compared to 55 nM *vs*. 1300 nM in the related ester **11**). In finding that the glycoside could be replaced with these alternative groups while preserving potency and selectivity, these compounds also had reduced total polar surface area (tPSA) and in the case of the azetidine-containing derivatives also had lowered cLogD_7.4_ [23].

**Figure 5:**
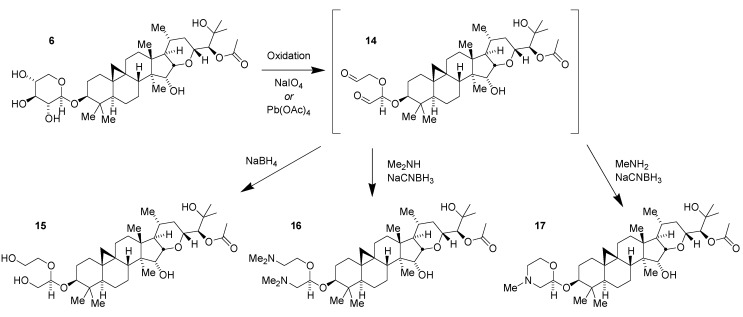
Synthesis of derivatives of compound **6** via dialdehyde **14**.

In another approach to modifying the natural product **1** to elaborate on SAR and generate new potent compounds no longer containing the large and polar glycoside, the ability to directly modify the sugar was exploited (Figure 5) [24]. Selective 1,2-diol-directed oxidation of the sugar in **6** affords the dialdehyde **14**. Direct reduction of the dialdehyde to the resulting tetraol **15** maintained potency though with no improvements in pharmacological properties. Double reductive amination with dimethylamine to obtain the corresponding diamine **16** resulted in a significant loss of activity. With methylamine it was possible to achieve a two-stage dual reductive amination to form the *N*-methyl morpholine **17**. This compound maintained potent pharmacology (Aβ42 IC_50_ = 130 nM) while improving physicochemical properties by lowering the tPSA to 98 Å^2^ and the hydrogen bond donor count to two. cLogP remained high at 4.5. Having established practical access to morpholine derivatives, it was possible to prepare an extensive SAR series of compounds which produced a diverse range of potent and selective compounds including morpholines modified with sulfonamide, urea and amide groups [24].

**Figure 6:**
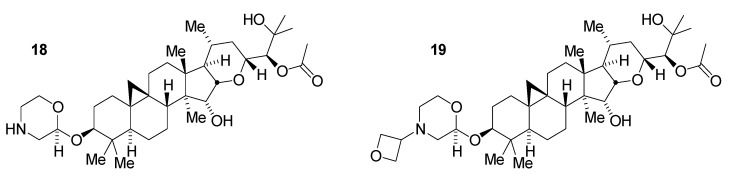
Morpholine derivatives of tetrahydropyran **6**.

The overall best compounds selected from this series were the parent morpholine **18** and the corresponding morpholino-*N*-oxetane derivative **19** (Figure 6). Particular attributes of these compounds, in addition to their potency and selectivity, included a promising balance of hydrogen bond donor number, tPSA, and lipophilicity. Inclusion of the basic morpholine was expected to assist in permeating the blood-brain barrier as well, in comparison with other compounds in this series. Preliminary pharmacokinetic studies in normal CD1 mice demonstrated sufficient CNS availability to allow study of PK/PD responses *in vivo* [25]. While having an improved profile in comparison with the native glycoside, these morpholine series compounds still required further optimization to achieve a more robust drugability profile.

### 2.3. Lead Optimization

**Figure 7:**
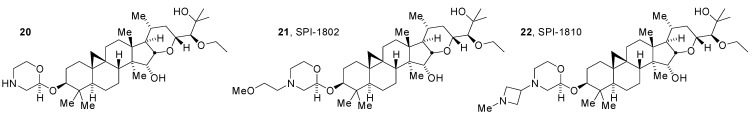
C24-*O*-Ethyl morpholines with improved microsomal stability.

Early lead compounds in the morpholine series still contained the C24 acetate. As with the initially isolated natural products, removal of this acetate to obtain the corresponding C24 alcohols uniformly lowered activity. The alcohols did, however, gain improved stability to incubation with human liver microsomes [25]. Attempts to obtain morpholino *N*-modified compounds in the presence of the C24 alcohol were uniformly unproductive at providing compounds with a potency approaching that of the acetates. In response to these observations, alternative modifications of the C24 hydroxyl group were evaluated resulting in the finding that the partially isosteric ethyl group would serve as an effective replacement for the acetyl group while providing substantially increased microsomal stability as in compounds **20**, **21** (SPI-1802) and **22** (SPI-1810) (Figure 7). While these GSMs demonstrated potent activity *in vitro* and in rodents [25,26] and had promising pharmacokinetic profiles in rodents, they also potently inhibited cytochrome P450 enzymes including CYP3A4. This property indicated a high potential to cause drug-drug interactions (DDIs) and therefore precluded consideration of these compounds as clinical development candidates [27]. Further optimization was required to minimize this potential for DDIs.

**Figure 8:**
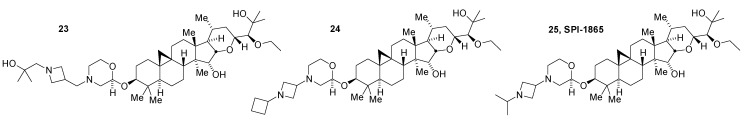
Morpholine derivatives with lower CYP inhibition.

Screening of *N*-modified 24-*O*-ethyl-3-*O*-morpholine series compounds for CYP inhibition revealed an inverse relationship between the combination of size and basicity of a substituent on the morpholine nitrogen and CYP inhibition (e.g., **20** > **21** > **22** at inhibiting CYP3A4) [27]. But, this was not a simple trend and computational modeling based on available structural data was used to develop a more detailed understanding of the available SAR with respect to CYP inhibition. This modeling effort suggested the synthesis of additional morpholine diamines. Among the compounds prepared to explore this molecular space, compounds **23**, **24**, and **25** (SPI-1865; Aβ42 IC_50_ = 75~110 nM) were chosen for further characterization based on maintaining potency, having lower levels of CYP inhibition, and a certain level of structural diversity (Figure 8) [27,28]. Pharmacokinetic studies in rats for these compounds showed low clearance, high volumes of distribution, a long half-life (79–129 h) and moderate to good bioavailability. All three compounds also had good brain availability in rats and lowered Aβ42. In normal and transgenic Tg2576 mice, **25** decreased brain Aβ42 and Aβ38 levels with little to no effect on Aβ40 [29]. Taken together, these data showed that SPI-1865 was orally bioavailable, brain penetrant, and effective at lowering Aβ42 in a dose responsive manner. Following 14-day rat safety studies to determine tolerated dose levels, **25** was selected as a development candidate based on its overall profile.

### 2.4. Gamma Secretase Inhibitors and Modulators—Comparative Pharmacology

Classic GSMs shift gamma secretase cleavage of APP from the site corresponding to Aβ42 to that of Aβ38, which is different from **25** that reduces both Aβ38 and Aβ42. To establish a better understanding of the properties of different classes of GSIs and GSMs, Satori compounds **21** and **22** were compared with structurally different compounds in a range of assays [26]. While GSIs inhibit global gamma secretase activity including inhibition of other physiologically important proteins such as Notch, GSMs do not affect Notch processing at concentrations that otherwise do not affect cell viability. GSIs tend to appear more potent in assays based on cell lines that over express APP, apparently due to differences in enzyme/substrate ratio [30]. This potency shift is not observed with GSMs based on aryl acetic acid NSAIDs, aryl imidazoles, or BCE [26]. The first two classes of compounds, with high aromaticity and logP in combination with low polar surface area, do not fit the profile of more typical marketed orally available drugs [26]. The Satori compounds such as **21**, **22**, and **25** have their own differences from defined properties associated with small molecule “drugability,” but are not excessively dissimilar from other marketed drugs derived from natural products [26]. Among the GSMs, differing structural types exhibit varying effects on the changes in the lengths of Aβ(1-x) that result; in the case of **21** and **22**, a reduction of Aβ38 was found in parallel with an increase of Aβ37. These data suggest the presence of more than one discrete allosteric binding site for GSMs within the gamma secretase complex [26,31,32]. While GSMs display potent activity *in vitro* as defined by IC_50_, they generally require much higher concentration *in vivo* to achieve similar levels of Aβ42 reduction. This observed difference from GSIs is not easily attributed simply to less than ideal properties such as high plasma protein binding or limited blood brain barrier permeability and may also include the ability to bind to components of gamma secretase such as presenilin 1 or bind to substrate, e.g., APP, even prior to formation of the active enzyme complex [26,33,34,35].

### 2.5. Scale-Up Chemistry

**Figure 9:**
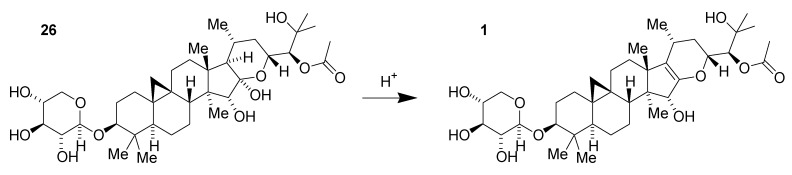
Acid-catalyzed dehydration of shengmanol **26** to obtain enol ether derivative **1**.

As with many natural product-derived compounds, scale-up chemistry had the potential to be quite challenging for these black cohosh-based GSMs since the amount of **1** present in extract is limited. However, early fractionation studies suggested that other compounds in the mixture could be transformed into useful intermediates. We promptly realized that very mild acid could catalyze the dehydration of shengmanol **26**, previously isolated from the extract, to the corresponding enol ether **1** (Figure 9).

**Figure 10:**
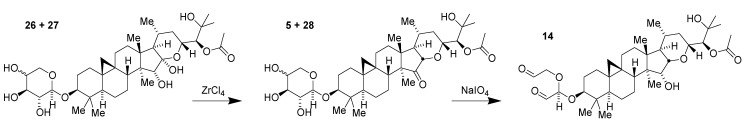
Conversion of shengmanol glycosides to key synthetic intermediate **14**.

This transformation had been anticipated as a potential step in the synthesis or semi-synthesis of such enol ethers and can increase their relative concentration in the plant extract. Additionally, xyloside **26** and the corresponding arabinoside **27**, which are significantly more abundant than **1**, can be harnessed for the preparation of the morpholine series derivatives (Figure 10). Additional compounds in the extract possibly contribute to the increased yield as well. A practical path to kilogram-scale synthesis of the key synthetic intermediate dialdehyde **14** thus emerged following optimization of bench-scale chemistry to be more suited to larger-scale manipulations [36].

A key component of this process was the use of liquid-liquid extraction of raw extract containing 2.5% of **26** and **27** to obtain a fraction enriched to a level of over 13% of **26** and **27** combined. This relatively unrefined mixture was then subjected to treatment with ZrCl_4_ to generate a mixture enriched in xyloside ketone **5** and its corresponding arabinoside **28**. Following workup of this reaction mixture, normal phase silica chromatography, and a final re-extraction, a highly enriched mixture of the desired ketones **5** and **28** was obtained. Selective 1,2-diol-directed oxidation of the glycosidic ketones then provided the single intermediate **14** for the preparation of the morpholine series derivatives.

From seven thousand kilograms of black cohosh root and rhizome biomass subjected to ethanolic extraction, 1330 kilograms of solids were obtained and subjected to further processing to obtain 5.1 kilograms of intermediate product containing over 94% of **26** and **27** [36] suitable for use as starting material for the kilogram-scale synthesis of **25** described in detail in [37]. Additional details and related information are contained in a series of patents and patent applications filed based on this work [38,39,40,41,42,43,44].

### 2.6. Development Status

Despite overcoming numerous metabolic and physicochemical liabilities in **1**, compound **25** and related backup compounds exhibited unexpected adrenal gland toxicity in rodent and monkey preclinical studies. This toxicity was reportedly due to a “physiochemical” problem unrelated to modulation of gamma secretase [45]. As a result of this toxicity, the Satori Pharmaceuticals GSM program, which had raised $47.3 million in institutional financing over nine years [46], was halted in May 2013 and the Company ceased operations [45].

## 3. Summary and Future Opportunities

Targeted screening was able to identify BCE as containing Aβ42 selective GSM activity. Bioassay-guided fractionation identified a series of active compounds including structurally novel shengmanol enol ethers including **1**, a potent and selective Aβ42-lowering GSM. Medicinal chemistry studies to prepare derivatives and analogs of **1** resulted in a broad range of semi-synthetic compounds culminating in the identification of **25** (SPI-1865) as a preclinical development candidate. Harnessing a convergent strategy to exploit multiple components of BCE for the synthesis of the key synthetic intermediate dialdehyde **14** allowed the kilogram-scale synthesis of **25**. Safety concerns identified during preclinical development precluded advancement into clinical testing. Additional study of the SAR relationships and pharmacology of these and related compounds may be useful to clarify their toxicity and lead to the development of alternative scaffolds for the synthesis of additional novel compounds of potential use as therapeutic agents.

Unaddressed questions related to this body of work are manifold. One interesting question is just how much of the most potent natural products identified in BCE are actually present in the plant itself as opposed to being generated during the extraction process? Are there additional compounds in the extract with the desired activity? The directed fractionation effort that isolated **1** did not fully explore other fractions of the extract with Aβ42-lowering activity. The biosynthetic pathways of these compounds are not well defined and given the distinct pharmacology associated with them this remains an interesting area of study. Black cohosh was chosen as a screening sample based on its long established and relatively safe use in traditional folk medicine. Other related plants contain the same and similar compounds that may provide additional sources of materials for pharmacological and medicinal chemical study.
